# Anti-ANGPTL3-FLD monoclonal antibody treatment ameliorates podocyte lesions through attenuating mitochondrial damage

**DOI:** 10.1038/s41419-022-05313-7

**Published:** 2022-10-13

**Authors:** Qianying Lv, Xinli Han, Jiajia Ni, Qianqian Ma, Rufeng Dai, Jiaojiao Liu, Jialu Liu, Yihui Zhai, Qian Shen, Li Sun, Haimei Liu, Jia Rao, Hong Xu

**Affiliations:** 1 Department of Nephrology, Children’s Hospital of Fudan University, National Pediatric Medical Center of CHINA, Shanghai, China; 2 Shanghai Kidney Development and Pediatric Kidney Disease Research Center, Shanghai, China; 3 Department of Rheumatology, Children’s Hospital of Fudan University, National Pediatric Medical Center of CHINA, Shanghai, China

**Keywords:** Focal segmental glomerulosclerosis, Target identification

## Abstract

Proteinuria, an indication of kidney disease, is caused by the malfunction of podocytes, which play a key role in maintaining glomerular filtration. Angiopoietin-like 3 (ANGPTL3) has been documented to have a cell-autonomous involvement in podocytes, and deletion of Angptl3 in podocytes reduced proteinuria in adriamycin-induced nephropathy. Here, we developed a monoclonal antibody (mAb) against ANGPTL3 to investigate its effects on podocyte injury in an ADR nephropathy mouse model and puromycin (PAN) induced podocyte damage in vitro. The mAb against the human ANGPTL3-FLD sequence (5E5F6) inhibited the binding of ANGPTL3-FLD to integrin β3. Treatment with the 5E5F6 mAb in ADR nephropathy mice mitigated proteinuria and led to a significant decline in podocyte apoptosis, reactive oxygen species (ROS) generation and mitochondrial fragmentation. In PAN-induced podocyte damage in vitro, the 5E5F6 mAb blocked the ANPGPLT3-mediated activation of integrin αvβ3 and Rac1, which regulated the mitochondrial homeostasis. Altogether, anti-ANGPLT3-FLD mAb attenuates proteinuria and podocyte lesions in ADR mice models, as well as PAN-induced podocyte damage, in part through regulating mitochondrial functions. Our study provides a therapeutic approach for targeting ANGPTL3 in proteinuric kidney disease.

## Introduction

Podocyte damage is an initial step in the nephrotic syndrome known as podocytopathy, represented by minimal change disease (MCD) or focal segmental glomerulosclerosis (FSGS) [1]. The podocyte is a terminally differentiated cell with distinct cell morphology that is primarily dependent on a highly dynamic underlying cytoskeletal network and that is critical for maintaining glomerular function and integrity in healthy kidneys [2]. Disruption of podocyte morphology in the form of foot process effacement or slit diaphragm remodeling leads to severe proteinuria and nephrotic syndrome (NS). Although immunosuppressive therapy is the most common treatment option for NS, not all patients respond to such therapy [3]. Additionally, there are growing concerns about the side effects of immunosuppressants and the nonresponse to them in podocytopathies [3]. Underlying the mechanism of podocytopathy helps to develop novel therapies for proteinuria.

Angiopoietin-like 3 (ANGPTL3) is a secreted protein with multiple functions involving in promoting neovascularization and hyperlipidemia. ANGPTL3 shares tertiary structural domains with angiopoietins, with N-terminal CCD (coiled-coil domain) and C-terminal FLD (fibrinogen-like domain). ANGPTL3-CCD is a key regulator of lipid metabolism [4]. ANGPTL3-FLD is thought to participate in angiogenesis by binding to integrin αvβ3 [5]. It is mainly expressed in the liver, with minimal expression in the kidney under physiological conditions [4]. ANGPTL3 was shown to be up-regulated in the glomerulus of patients with NS (including minimal change disease, focal segmental glomerulosclerosis, IgA nephropathy and membranous nephropathy) as well as in cultured podocytes treated with adriamycin (ADR) or puromycin amino nucleoside (PAN) [6–8]. Angptl3 deletion significantly reduced proteinuria in ADR nephropathy mice model and protected podocytes from apoptosis caused by ADR or PAN in vitro [6, 7]. Additionally, we demonstrated that ANGPTL3 may interact with podocyte-expressed integrin αvβ3 [7, 9] leading to the activation of integrin-mediated cellular signaling pathways in cultured podocytes [9]. FLD may be the key component of ANGPTL3 implicated in podocyte injury since it is the binding site of ANGPTL3 and integrin αvβ3. Therefore, targeting the ANGPTL3-FLD might be a novel therapeutic strategy for proteinuria.

Mitochondrial dysfunction is one of the key processes involved in podocyte injury and loss. Deletions in mitochondrial DNA (mtDNA) have been noted in roughly 60% of primary FSGS patients [10]. ADR-induced podocyte injury is also caused by mtDNA variants and a decrease in mtDNA copy numbers [11]. Meanwhile, mitochondrial dysfunction coincides with its morphology changes. A balance of mitochondrial fusion and fission contributes to the maintenance and optimization of mitochondrial function [12]. ANGPTL3 was shown to participate in mitochondrial function and morphology changes in adipose tissue [13, 14]. Thus, it suggests that ANGPTL3-related mitochondrial dysfunction plays an essential role in podocytopathy.

Based on this hypothesis, we immunized mice with the recombinant protein of human ANGPTL3 to generate an anti-ANGPTL3-FLD monoclonal antibody (5E5F6 mAb) and investigated the potential effect of the mAb in podocytopathy. Our data demonstrated the agonistic anti-ANGPTL3 mAb attenuated podocyte injury via reducing mitochondrial dysfunction in podocytes.

## Material and method

### Antibodies and regents

The primary antibodies used in this study targeted ANGPTL3 (Abcam, #ab126718), WT1 (Abcam, #ab89901), cleaved caspase3 (Affinity, #Ab-AF7022), caspase 8 (Proteintech, #13423-1-AP), caspase 9 (Proteintech, #10380-1-AP), BAX (Proteintech, #50599-2-Ig), Bcl2 (Proteintech, #12789-1-AP), GAPDH(Affinity, #AF7021), Anti-Integrin β3-AP5 antibody (kerafast, #EBW107), NRF1 (Proteintech, 12482-1-AP), MFN1(Proteintech, 13798-1-AP), mitochondrial transcription factor A (TFAM, Proteintech, 22586-1-AP), DRP1(Proteintech, 12957-1-AP), Rac1-GTP (NewEastBio, #26903), PINK1(Proteintech, 23274-1-AP), Parkin (Proteintech, 14060-1-AP), p62 (Affinity, #AF5834), COX IV (CST, #4850), LAMP1(CST, #15665). The reagents used in this study including DCFH-DA (Beyotime, #S0033M) assay kit, mitochondrial membrane potential (Beyotime, #C2006) assay kit, MitoTracker Red (Beyotime, #C1035), MitoSOX Red (Invitrogen, #M36008), the genomic DNA preparation kit (TIANGEN, #DP304), the Mitochondrial Respiratory Chain Complex I, Complex II, Complex III, Complex IV and Complex V activity (Solarbio, BC0515, BC3235, BC3245, BC0945, BC1445) assay kit, the PE Annexin-V Apoptosis Detection kit I (BD Bioscience, #559763), the MTT cell proliferation and cytotoxicity assay kit (Nanjing Jian Cheng Bioengineering Institute, #G020-1-1), creatinine detection kit (Nanjing Jian Cheng Bioengineering Institute,#C011-1-1) and total cholesterol detection kit (Nanjing Jian Cheng Bioengineering Institute, #A111-1-1), mouse urinary albumin detection kit (Chondrex, #3012).

### Generation of anti-ANGPTL3-FLD monoclonal antibody

Mouse hybridoma antibodies against the human ANGPTL3-FLD sequence were generated by immunizing BALB/c mice with human ANGPTL3 recombinant protein. According to their protocols, immunization, hybridoma production, and initial screening were conducted at GenScript Biotech (GenScript USA, Inc.). Enzyme-linked immunosorbent assays (ELISAs) were used in the screening of hybridomas against the human ANGPTL3-FLD sequence. The positive clones (40 clones) were further screened by immunoblotting using cell lysates from Escherichia coli stably expressing mouse ANGPTL3-FLD. Twenty clones were positive for immunoblotting. The top 4 clones that showed strong immunoreactivity in all tests were subjected to small-scale antibody production. The antibody transient expression analysis indicated that 5E5F6 had the highest expression among the four antibodies. Therefore, 5E5F6 (isotype IgG1/Kappa) was chosen for functional assessment. According to their protocols, antibody production, purification, subcloning, and isotyping were conducted at GenScript Biotech.

### Functional characterization of 5E5F6 in vitro

#### BIAcore analysis

Surface plasmon resonance (SPR) experiments were performed using a Biacore T200 machine at 25 °C. HBS-EP was used as the running buffer. In these measurements, the ANGPTL3-FLD recombinant protein was coupled on a CM5 chip at pH 5.0 using an amine coupling kit (GE Healthcare, USA). Samples at a 6.25–800 nM concentration were captured on the second flow cell at a flow rate of 30 μl/min. A dilution series of the 5E5F6 monoclonal antibody was passed through both flow cells at 30 μl/min to record the association phase (180 s). The dissociation phase was monitored for 480 s and triggered by replacing the sample solution with HBS-EP. After each cycle, the sensor surface was regenerated with a short treatment using ten mM glycine-HCl (pH 2.1). Biacore T200 evaluation software (GE Healthcare, USA) was used to record and analyze the binding kinetics using the 1:1 binding model.

#### Computer-guided modeling

Computer-guided modelling was performed as described [15]. The integrin αvβ3-b1 domain and ANGPTL3-FLD protein of Homo sapiens were retrieved from the Protein Data Bank. The structure of the 5E5F6-Fab was modelled on the Swiss-Model website. The protein structure was modelled by the homology modelling method using the prime program of the Schrödinger software suite. Loops were refined, and verification was performed by the protein refinement program of Schrödinger software. A modeled protein structure was used to identify effective binding sites based on structural and physical properties using the sitemap program of Schrödinger software.

#### ELISA and competitive ELISA

A high-affinity microplate (Costar, USA) was coated with 1 μg/ml integrin αvβ3 at four °C overnight. Serial biotin-labeled ANGPTL3-FLD concentration gradients were added to the wells at room temperature for two h. After washing, peroxidase-labeled streptavidin was added to the plate and incubated for one h at room temperature. TMB was used as the substrate for the color reaction, and the light absorbance was measured at 450 nm. A competitive ELISA was used to evaluate the activity of the 5E5F6 monoclonal antibody that inhibited the interaction between ANGPTL3-FLD and its ligand integrin αvβ3. High-affinity microplates were coated with integrin αvβ3. Biotin-labeled ANGPTL3-FLD was diluted to a saturated concentration (0.16 μg/ml), and the 5E5F6 mAb was serially diluted with the configured ANGPTL3-FLD biotin solution. Serial 5E5F6 mAb concentration gradients were added to the wells at room temperature for two h. The substrate and enzyme described above were used to test the blocking signal.

### Mouse model

**Fig. 1:**
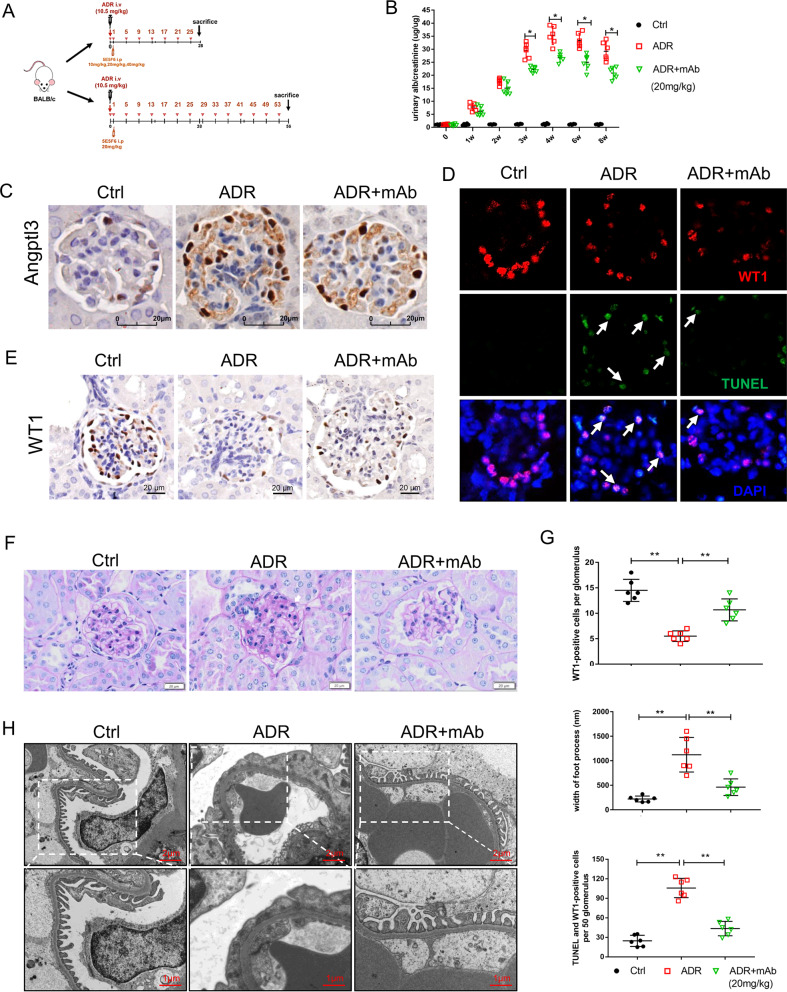
In ADR-induced nephropathy mice, anti-ANGPTL3-FLD monoclonal antibody (5E5F6) preserve podocytopathy. **A** Schematic figure of experimental design and 5E5F6 dosing plan. **B** Evaluation of proteinuria at 0 to 8th weeks. Figures show individual data of each animal and the mean ± SEM of the different groups at each time point (*n* = 6 per group). **C** The expression of Angptl3 in glomeruli of mice was detected by immunohistochemistry staining. **D** TUNEL staining in different groups. A total of 50 glomeruli per kidney were calculated. White arrow: Both TUNEL and WT1-positive staining cells. Original magnification 400×. **E** Representative immunohistochemistry images against podocyte marker Wilms tumor protein1 (WT1; brown stained nuclei) for control mice, ADR nephropathy mice, and 5E5F6 mAb-treated ADR nephropathy mice (ADR + mAb) Magnification ×400. Bars = 20 μm. **F** Representative images of light microscopy of kidney sections of control mice, ADR nephropathy mice (ADR), and 5E5F6 mAb-treated ADR nephropathy mice (ADR + mAb), stained with periodic acid–Schiff (PAS) in 8w. Original magnification: ×400, Bar = 20 μm. Arrows indicate tuft adhesion. **G** Average WT1-positive cell numbers per glomeruli, the width of foot process detected by transmission electron microscopy (TEM), TUNEL-positive and WT1-positive cells per 50 glomeruli were quantified. Figures show individual data of each animal and the mean ± SEM of the different groups (60 glomeruli per group in immunobiological staining; *n* = 6, *p < 0.05, **p < 0.01). **H** Representative images of the TEM analysis show significant damage to podocyte foot processes in ADR nephropathy mice, whereas relatively normal foot processes were present in the 5E5F6 mAb-treated ADR nephropathy mice (ADR + mAb). Upper row, scale Bar 2 μm, original magnification 3400. Bottom row, scale Bar 1 μm, original magnification 6800.

Animal experiments were approved by the Animal Ethics Committee of the Children’s Hospital of Fudan University (NO. 2020_235) and conducted in accordance with the recommendations of the Guidelines for Ethical Conduct in the Care and Use of Animals. BALB/c mice (6–8 weeks old) were obtained from Shanghai SLAC Laboratory Animal Co., Ltd. All the mice had access to water and food ad libitum, and were randomly divided into the control (ctrl) group, 5E5F6 mAb (mAb) group, adriamycin nephropathy (ADR) group, ADR + mAb group, and the non-specific IgG group. Six mice were included in each group. Saline or adriamycin at a single dose of 10.5 mg/kg was injected into each mouse via tail vein injection on day 0 [16]. Mice in the mAb group and ADR + mAb group received intraperitoneal (i.p.) injection of 5E5F6 mAb (10 mg/kg, 20 mg/kg or 40 mg/kg for 4-week or 20 mg/kg for 8-week) every four days from day 1. Mice in the non-specific IgG group received the same dose of non-specific IgG injection. A pilot experiment was performed to select an optimal 5E5F6 dose. The experimental design and 5E5F6-dosing plan are presented in a schematic Figure (Fig. 1A).

#### Biochemical indexes measurement and urine analysis

Mice were sacrificed on day 28th and day 56th, the serum samples were collected for biochemical measurements. The urine samples were collected before and 1, 2, 3, 4, 6 and 8 weeks after the intervention. Urinary albumin concentration and urinary creatinine were detected by an ELISA kit for mouse albumin (Chondrex, Inc) and a creatinine assay kit (Nanjing Jiancheng Bioengineering Institute).

#### Histological examination

The kidney tissue was fixed with 4% paraformaldehyde, embedded in paraffin, and made into paraffin sections. The paraffin sections were stained with PAS to detect the morphological changes or used for immunohistochemical staining. The calculation of the number of WT1-positive cells per glomerulus has been described previously [17]. Frozen sections of kidney tissue were fixed with 4% paraformaldehyde, phosphate-buffered saline containing 0.5% Triton X-100 was used for permeabilization, and then the samples were incubated with anti-WT1 and anti-AP5 antibodies. The primary antibodies were revealed with the appropriate Alexa Fluor 488- and Cy3-conjugated secondary antibodies. Immunofluorescence staining was visualized using a laser scanning confocal microscope (Leica, Wetzlar, Germany). Transmission electron microscopy (TEM) sample handling and detection were performed as follows. In brief, the kidney tissue and cultured podocyte samples were first prefixed in 2.5% glutaraldehyde. Then, the samples were washed in PBS (0.01 M) and postfixed with 1% osmium acid. After gradient dehydration in ethanol and acetone, the samples were embedded. Five glomeruli were randomly selected from each mouse, and 10 electron micrographs were taken from each glomerulus. ImageJ software (National Institutes of Health, NIH, Bethesda, MD) was used to analyze the TEM images of kidney tissue and cultured podocytes.

#### Cell culture and treatment

Conditionally immortalized human podocytes were cultured as described previously [18]. The podocyte cells were grown on collagen-coated culture dishes at 33 °C and 5% CO_2_, and were differentiated by thermo switching to 37 °C. Podocyte injuries were developed using puromycin aminonucleoside (PAN, 50 μg/ml) for 24, 48 h. In the 5E5F6 treatment group, podocytes were pretreated with 5E5F6 (100 ng/ml, 500 ng/ml) for 1 h before treatment with PAN.

#### Flow cytometry

The apoptosis rate of podocytes was measured by flow cytometry using a PE-conjugated annexin V/7-AAD kit. The experimental procedure was conducted according to the manufacturer’s instructions.

#### MTT cell viability assay

Podocyte viability was determined using an MTT cell proliferation and cytotoxicity assay kit. The experiment was performed in accordance with the manufacturer’s instructions.

#### Immunocytochemistry

Podocytes on the coverslips were rinsed with PBS and fixed with 2% paraformaldehyde, phosphate-buffered saline containing 0.5% Triton X-100 was used for permeabilization, and then the samples were incubated with anti-AP5, anti-Cox IV and anti-LAMP1 antibodies. The primary antibodies were revealed with the appropriate Alexa Fluor 488-, Alexa Fluor 555- and Cy3-conjugated secondary antibodies.

#### Western blot analysis

Western blots were performed as previously described. In short, the same amount of total protein (25 μg per well) was subjected to SDS-PAGE, and then immunoblotting was performed on NC membranes. The primary antibodies used are listed in Antibodies and Reagents. ImageJ software was used to quantify the grayscale value of the band.

#### mtDNA measurement

Based on the manufacturer’s protocol, whole genomic DNA was extracted, and the real-time PCR was used for the relative quantification of mtDNA copy number. DNA primers were designed to detect Cyt b and cytochrome c oxidase subunit II (COII) as markers for mtDNA and 18S rRNA as a marker for nuclear DNA (nDNA). The relative copy number of mtDNA is represented by the ratio of mtDNA to nDNA (mtDNA: nDNA), which was calculated by the 2-ΔΔCT method.

#### MitoSOX staining

The MitoSOX Red 5 mM stock solution was diluted with Hank’s balanced salt solution (HBSS) to a final concentration of 5 μM. After treatments, podocytes were collected and incubated with MitoSOX Red for 10 min in the dark at 37 °C. The cells were then washed three times with HBSS and observed using a laser scanning confocal microscope (Leica, Wetzlar, Germany).

#### MitoTracker staining

MitoTracker Red CMXRos working fluid (100 nM) was prepared. After treatment, the cell culture solution was removed, and the prepared MitoTracker Red CMXRos working solution was used to incubate the podocytes at 37 °C for 30 min in the dark. Podocytes were observed with a laser confocal microscope.

#### Mitochondrial membrane potential detection

The mitochondrial membrane potential of podocytes was detected using a mitochondrial membrane potential assay kit with JC-1, and the experiments were performed as described previously. Briefly, JC-1 working solution was prepared according to the manufacturer’s instructions and used for podocyte incubation in a 37 °C cell incubator for 20 min. After incubation, the supernatant was aspirated, and the cells were washed twice with JC-1 staining buffer (1X). The podocytes were observed using a laser confocal microscope.

#### Reactive oxygen species (ROS) production detection

Briefly, podocytes and frozen fresh kidney sections were loaded with ten μM DCFH-DA and incubated for 20 min at 37 °C in the dark. After incubation, podocytes and kidney sections were subsequently washed with PBS and visualized using a laser confocal microscope.

#### Mitochondrial respiratory chain complex activity assay

Mitochondria wasisolated from podocytes, and the activity of mitochondrial respiratory chain complex I, complex II, complex III, complex IV and complex V was measured using the Mitochondrial Respiratory Chain complex activity assay kit according to the manufacturer’s instructions. Briefly, adding the mitochondrial homogenates of each group to the respective reaction buffer and the activity of complex I, complex II, complex III, complex IV, and complex V was determined at 340 nm, 605 nm, 550 nm (for complex III and complex IV) and 660 nm in the spectrophotometer, respectively.

### Statistical analysis

All data were presented as mean ± SD. Statistic analysis was performed with GraphPad Prism 7.0 (GraphPad Software. San Diego, CA). Two way-ANOVA was used to determine differences between groups at different time points. One way-ANOVA was used to determine differences between groups at a single time point. Student’s *t*-test was used to assess differences between samples. *P* values < 0.05 were considered significant (*p < 0.05 and **p < 0.01).

## Results

### Upregulation of Angptl3 in the kidneys of the adriamycin-induced mouse model was associated with podocyte damage

Based on the previous work on ANGPTL3 and podocytopathy, we profiled the Angptl3 expression in the ADR-induced mouse model until 8 weeks (Fig. 1A–C). Diffuse foot process fusion of podocytes was observed, coinciding with highly expressed Angptl3 on podocytes (Fig. 1G, H). Hypercholesterolemia, hypoalbuminemia, elevated creatinine levels and body weight loss were recorded in ADR-treated mice (Supplementary Fig. 3).

### Generation of anti-human ANGPTL3-FLD monoclonal antibody

**Fig. 2:**
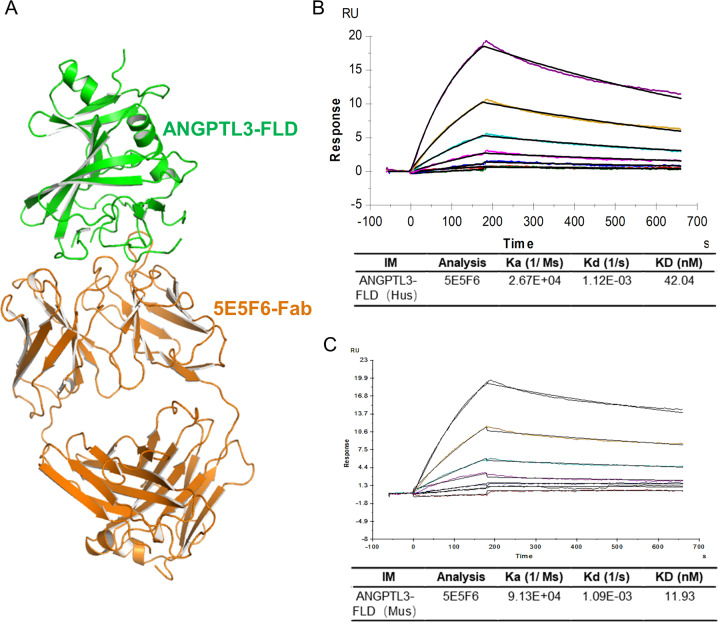
Characterization of the anti-ANGPTL3-FLD monoclonal antibody (5E5F6). **A** The ribbon structure of the human ANGPTL3-FLD and 5E5F6-Fab complex based on the computer-guided modeling and docking methods. **B** SPR sensorgram depicting the interaction of immobilized human ANGPTL3-FLD with 5E5F6. Rate constants (ka and kd) were determined by kinetic fitting of the sensorgrams using a one-to-one binding equation, and equilibrium dissociation constant (KD) values were determined by calculating kd/ka. The KD values of human ANGPTL3-FLD to 5E5F6 was 42.04 nM. **C** SPR sensorgram depicting the interaction of immobilized mouse ANGPTL3-FLD with 5E5F6. Rate constants (ka and kd) were determined by kinetic fitting of the sensorgrams using a one-to-one binding equation, and equilibrium dissociation constant (KD) values were determined by calculating kd/ka. The KD values of mouse ANGPTL3-FLD to 5E5F6 was 11.93 nM.

Previous work has showed that siRNA treatment of Angptl3 reduced the proteinuria and hypertriglyceridemia in a rat model with puromycin-induced NS, indicating that ANGPLT3 is a potential target for podocytopathy [6, 7]. As the ANGPTL3-FLD domain is primarily involved in podocyte damage, mouse hybridoma antibodies against the human ANGPTL3-FLD sequence were tested. ELISA against the ANGPTL3-FLD (Homo) and western blotting with Angptl3-FLD (mouse) were used to screen the hybridomas. A total of 20 positive clones were tested. Four out of the twenty were selected for subcloning and expansion. These subclonal hybridomas were screened using the above methods. Finally, the clone 5E5F6 was chosen for further functional assessment. The monoclonal antibody clone 5E5F6 demonstrated the most powerful biological activity and binding activity, was used for this study. The molecular ribbon structure of human ANGPT3-FLD and 5E5F6-Fab complex was built through the computer-guided modeling and docking methods (Fig. 2A). The BIAcore KD value of 5E5F6 was 42.04 nM against ANGPTL3-FLD (Homo) and 11.93 nM against Angptl3-FLD (mouse) (Fig. 2B, C).

### Effect of 5E5F6 mAb in ADR-induced nephropathy mice model

To assess the possible renoprotective effect of 5E5F6 mAb in vivo, we administrated it to the mice with ADR-induced nephropathy (Fig. 1A), which is characterized by podocyte impairment, glomerular sclerosis and tubulointerstitial fibrosis [19]. Treatment of mice with various doses of the 5E5F6 mAb resulted in a dosedependent drop of proteinuria (Fig. 1B). At the fourth week after treatment, the urine protein level was reduced by ~22.7% and ~36.53% in ADR mouse model treated with a dose of mAb 20 mg/kg or 40 mg/kg. Proteinuria was dropped by ~28.61% in ADR model mice with a 20 mg/kg dosage of mAb after 8 weeks of follow-up (Supplementary Fig. 3, Fig. 1B). The 5E5F6 mAb effectively relieved hypercholesterolemia, hypoalbuminemia, elevated creatinine levels, and body weight loss in ADR-treated mice (Supplementary Fig. 3).

Periodic acid-Schiff (PAS) staining revealed podocyte vacuolar degeneration in the ADR mouse model at the 4th week and glomerular tuft adhesion at the 8th week. On the other hand, the kidney lesions in the 5E5F6 mAb-treated group were minor (Fig. 1F, Supplementary Fig. 4). Ultrastructural analysis using TEM revealed foot process broadening and effacement lesions in podocytes in the ADR-treated group, which was significantly alleviated in the ADR + mAb group (Fig. 1G, H). Furthermore, podocyte loss was assessed using the known podocyte marker Wilms-tumor 1 (WT1) (Fig. 1E). We illustrated that the number of WT1-positive cells per glomerulus was markedly reduced in the ADR-treated mice, and the 5E5F6 mAb significantly restored the number of WT1-positive cells (Fig. 1E, G). To detect podocyte apoptosis in glomeruli further, double immunofluorescence labeling with WT1 and TUNEL (TdT-mediated dUTP nick end labeling) were conducted. TUNEL was shown strongly stained in the podocytes of ADR-treated mice, but they were faintly stained in the ADR + mAb group (Fig. 1D, G).

### Effect of 5E5F6 mAb on PAN-induced podocyte apoptosis

**Fig. 3:**
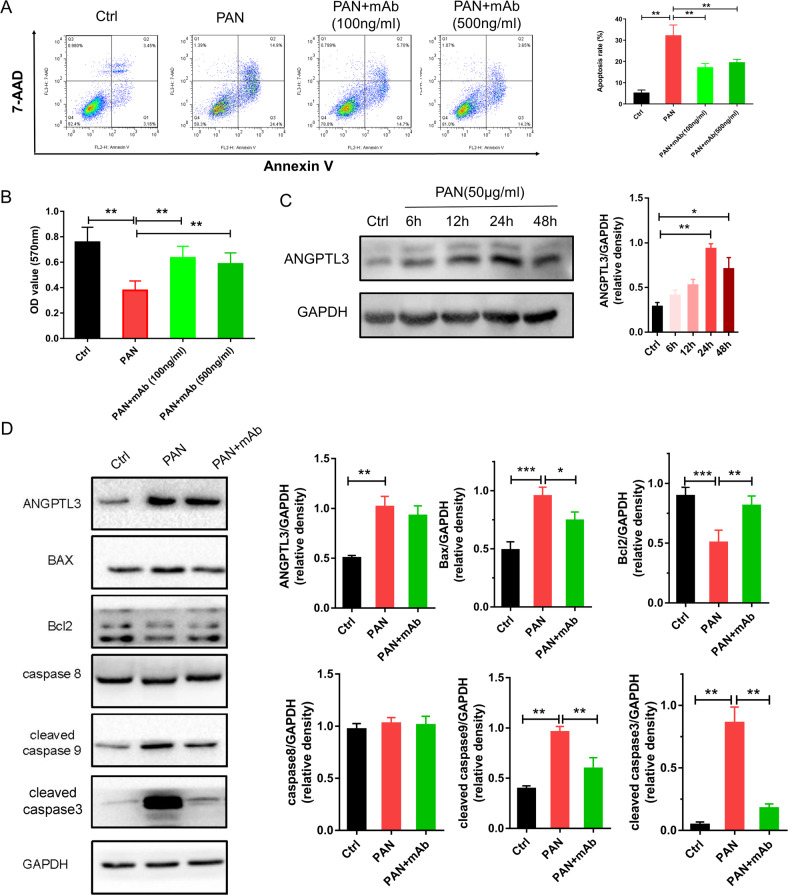
Effect of 5E5F6 mAb on PAN-induced podocyte apoptosis. **A** Flow cytometry analysis of apoptosis in podocytes incubated with different media and quantification of these results (*n* = 3). **B** Cell viability of podocytes was analyzed using an MTT assay following different treatments (*n* = 3). **C** Western blot assay showed the expression of ANGPTL3 at different time points in human podocytes after PAN treatment. *p < 0.05, **p < 0.01 compared with the Ctrl group. **D** Relative expression of ANGPTL3, BAX, Bcl2, caspase 8, cleaved caspase 9 and cleaved caspase3 in different groups (*n* = 3). *p < 0.05, **p < 0.01 compared with the PAN group.

To validate the function of 5E5F6 mAb in the PAN-induced podocyte apoptosis, flow cytometry with Annexin V/7-AAD double staining was performed. When compared to that of the PAN-treated group, the 5E5F6 mAb significantly reduced the proportion of early or late apoptotic cells (Fig. 3A). It did not show a significant change in the apoptotic ratio between the treatment groups with different doses of 5E5F6 mAb (*p* > 0.05). The 5E5F6 mAb improved podocyte survival following PAN treatment, as confirmed by MTT assay (Fig. 3B). ANGPTL3 expression was also examined in human immortalized podocytes at various time periods (6 h, 12 h, 24 h, 48 h) following PAN treatment (50 ug/ml). The expression level of ANGPTL3 increased significantly after 24 h or 48 h of PAN treatment and peaked at 24 h (Fig. 3C, Supplementary Fig. S6), while 5E5F6 mAb reversed the effect of PAN-induced ANGPTL3 expression (Fig. 3D, Supplementary Fig. S6).

The extrinsic (the death receptor) and intrinsic (mitochondrial) pathways were examined in PAN-induced podocyte damage. The Bcl-2 family of proteins, which includes Bcl-2 homology (BH) domains, pro-apoptotic members such as BAX and anti-apoptotic members such as Bcl-2, control the mitochondrial pathway of apoptosis [20]. In PAN-treated podocytes, apoptotic signaling molecules BAX, cleaved caspase9, cleaved caspase3 were significantly enhanced, whereas Bcl2 was depleted. The 5E5F6 mAb restored the Bcl2 expression while suppressing BAX, cleaved caspase9 and cleaved caspase3. No significant change in the expression of caspase 8 was observed (Fig. 3D, Supplementary Fig. S6, Supplementary Fig. S7).

### 5E5F6 mAb inhibited integrin β3 activation and ROS generation in podocytes

**Fig. 4:**
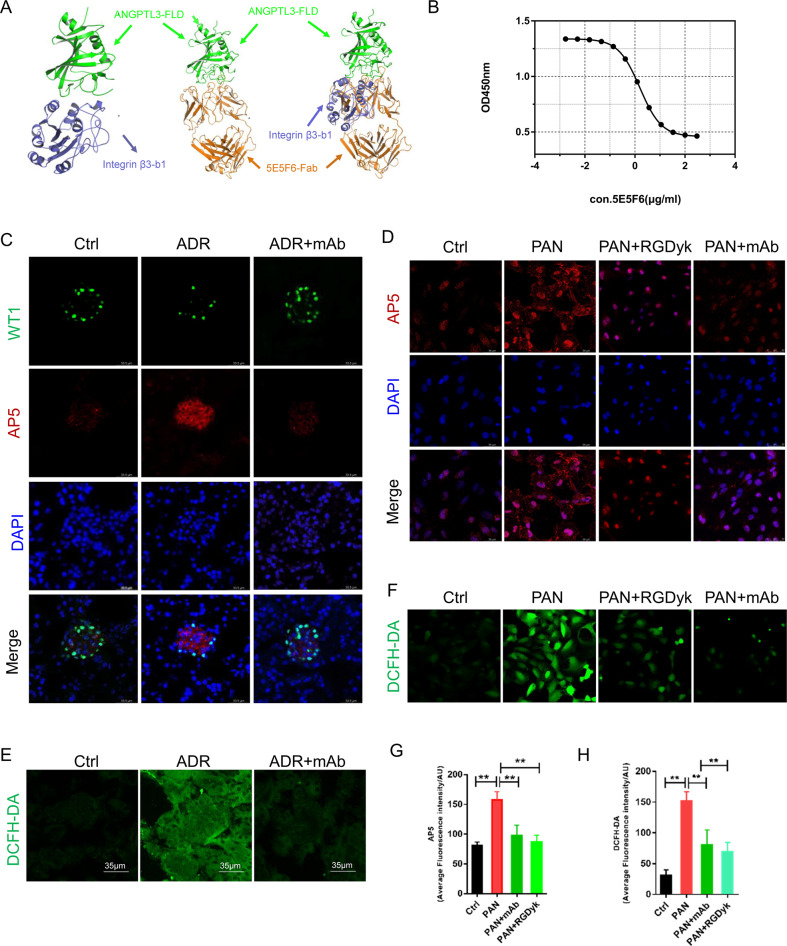
5E5F6 mAb reduced integrin β3 activation and ROS production in the podocyte. **A** The ribbon structure of the human ANGPTL3-FLD and integrin β3-b1 domain complex, human ANGPTL3-FLD and 5E5F6-Fab complex, human ANGPTL3-FLD, 5E5F6-Fab and integrin β3-b1 domain based on the computer-guided modeling and docking methods. **B** Analysis of the activity of 5E5F6 blocking the binding of ANGPTL3-FLD to Integrin αvβ3 by competitive ELISA assay, the EC50 is 1.57 ug/ml. **C** Immunofluorescence analysis of kidney tissue in different treatment groups. Kidney tissue were stained with AP5 antibody that recognizes the active form of integrin β3 and WT1 antibody to mark podocytes. Magnification in ×400. **D** Representative images showing AP5 staining in podocytes from different groups. **E** Fresh renal cortical tissues were obtained from the different treated mice and prepared as frozen cryostat sections for staining DCFH-DA (green), a marker of ROS. **F** Representative images of podocytes stained with DCFH-DA. **G** Average fluorescence intensity representing the level of integrin β3 activation detected. Data represent measurements of >50 cells and are plotted as means ± SD (*n* = 3). **H** Average fluorescence intensity representing the level of DCFH-DA detected. Data represent measurements of >50 cells and are plotted as means ± SD (*n* = 3). *p < 0.05, **p < 0.01 compared with the PAN group.

The activation of integrin αvβ3 in glomeruli and podocytes has been documented following ADR or PAN treatment [21, 22]. Previous studies found that the binding of podocyte-oriented ANGPTL3 to integrin αvβ3 might cause podocyte injury and proteinuria [7]. In this study, based on the binding site of integrin αvβ3 [23] and computer-guided modeling and docking for the complex of ANGPTL3-FLD and integrin β3-b1 domain, the 5E5F6 mAb was shown to compete for Integrin αvβ3-binding to ANGPTL3-FLD (Fig. 4A). Furthermore, a competitive ELISA revealed that 5E5F6 mAb efficiently blocked the binding of ANGPTL3-FLD to integrin β3 (Fig. 4B), with an EC50 of 1.57 ug/ml. Hence, we anticipated that inhibiting integrin β3 activation in podocytes would minimize podocyte damage and proteinuria. An immunofluorescence analysis of kidney tissue was performed, using an AP5 antibody that specifically recognizes the active form of integrin β3. ADR treatment resulted in substantial activation of Integrin β3, whereas the 5E5F6 mAb significant attenuated the activation of integrin β3 (Fig. 4C). AP5 labeling of podocytes in vitro demonstrated that integrin β3 was strongly activated by PAN, which was blocked by 5E5F6 mAb, as shown by the average level of fluorescence intensity (Fig. 4D, G). ROS generation was implicated in ADR or PAN-induced podocyte injury [24, 25], and the activation of integrin αvβ3 was involved in ROS production via small GTPases signaling [26, 27]. Thus, we investigated the ROS production in kidney tissue and podocytes. ADR, as an effective pro-oxidant, generates severe oxidative stress in the glomeruli and renal tubules, as seen by the green fluorescence of the DCFH-DA fluorescent probe. The 5E5F6 mAb reduced DCFH-DA fluorescence and oxidative stress in the glomeruli of ADR-damaged kidneys (Fig. 4E). Moreover, following 24 h of PAN exposure, the intracellular ROS production increased considerably in podocytes. Along with the inactivation of integrin β3 induced by 5E5F6 mAb, a substantial reduction in ROS was observed (Fig. 4F, H). This was confirmed by the pre-treatment with RGDyk, which can also inhibit the activation of integrin β3 and reduce ROS generation in PAN-induced podocyte damage (Fig. 4D, F, H), indicating that the integrin activation is involved in PAN-induced ROS production.

### 5E5F6 mAb restored mitochondrial dysfunction in podocytes

**Fig. 5:**
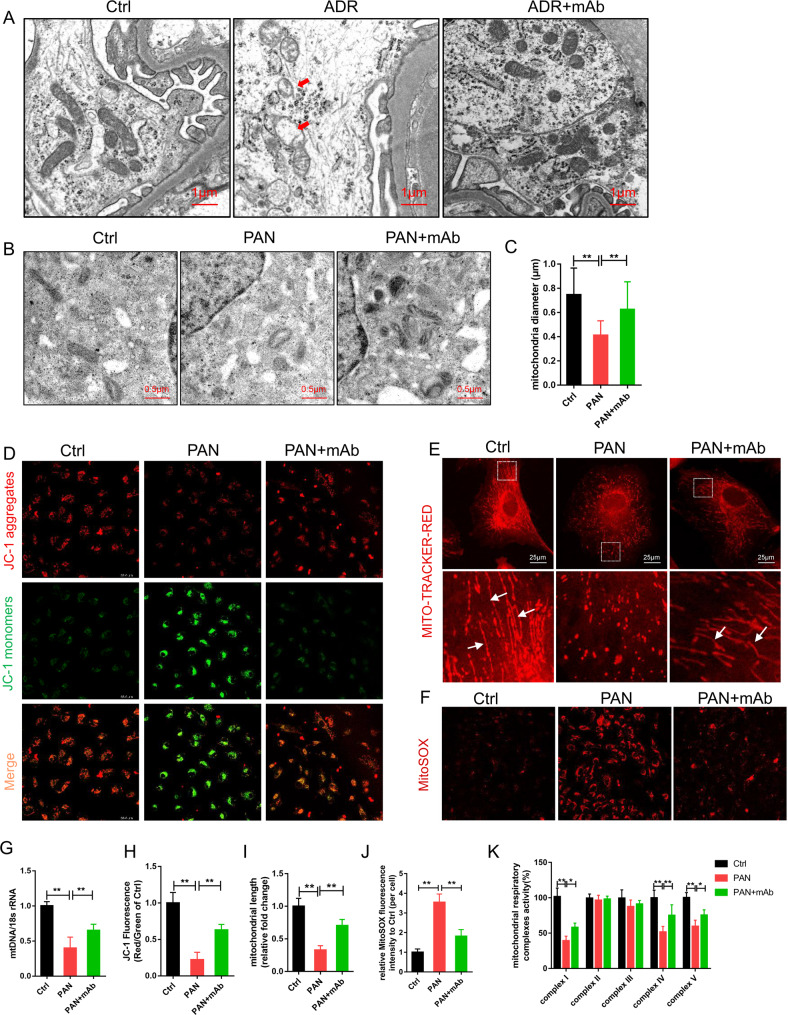
5E5F6 mAb restored mitochondrial dysfunction in podocytes. **A** Representative TEM images of glomeruli showing mitochondrial morphology in podocytes of kidney sections, mitochondria swelling, vacuolization, and cristae fragmentation in podocytes were observed in ADR nephropathy mice. Arrows indicate representative mitochondria. Bar = 1 μm, original magnification 6800. **B** Representative TEM images showing mitochondrial morphology. Bar = 0.5 μm, original magnification 13,600. **C** The mitochondrial diameter in cultured podocytes with different treatments was quantified. **D** Representative images of podocytes stained with JC-1. **E** Mito-Tracker staining was used to assess the mitochondrial morphology in podocytes. Elongated mitochondria were observed in healthy untreated podocytes (arrows), shortened fragmented mitochondria were noted in podocytes injured by PAN. Treatment with 5E5F6 mAb restored the mitochondrial morphology. Scale bar 25 μm. **F** Representative images of podocytes stained with MitoSOX. **G** Relative mitochondrial DNA copy number (mtDNA:18 s rRNA) in podocytes. The results were normalized to the ratio of mtDNA: 18 s rRNA of healthy untreated podocytes. **H** JC-1 fluorescence (red-to-green ratio) showing changes in fluorescence intensity in podocytes with different treatments were quantified. JC-1 fluorescence was normalized with the red-to-green ratio of podocytes in the Ctrl group. **I** The mitochondrial length in podocytes with different treatments were quantified. The results were normalized to the mitochondrial length of healthy untreated podocytes. Data represent measurements of >50 cells and are plotted as means ± SD (*n* = 3). *p < 0.05, **p < 0.01 compared with the PAN group. **J** MitoSOX fluorescence intensity in podocytes with different treatments were detected. Relative fluorescence intensity per cell was quantified versus control. Data represent measurements of >50 cells and are plotted as means ± SD (*n* = 3). *p < 0.05, **p < 0.01 compared with the PAN group. **K** The activity of mitochondrial respiratory chain complexes in different groups (*n* = 3). *p < 0.05, **p < 0.01 compared with the PAN group.

ROS have been identified as triggers of mitochondrial defects and cell apoptosis in multiple organs, including the kidneys [28]. As a result, we explored the morphology and function of mitochondria in podocytes. TEM revealed the mitochondrial damage in podocytes from ADR mouse models, which was mitigated by the 5E5F6 mAb, as demonstrated by changes in cristae number and size of mitochondria (Fig. 5A). PAN-treated podocytes had a higher proportion of fragmented mitochondria in vitro. By preserving the shape and diameter of mitochondria in podocytes, the 5E5F6 mAb pre-treatment restored PAN-induced mitochondrial defects (Fig. 5B, C). PAN-induced mitochondrial fission was greatly reduced by the mAb (Fig. 5E, I). Maintaining the mitochondrial DNA (mtDNA) copy number is critical for its function [29]. The 5E5F6 mAb also rescued the mtDNA copy number (mtDNA:18 s rRNA) in PAN-treated podocytes (Fig. 5G). The drop in mitochondrial membrane potential (MMP) induced by PAN was detected by JC-1 fluorescence, which was attenuated by the 5E5F6 mAb pretreatment in podocytes (Fig. 5D, H). MitoSOX labeling revealed that the 5E5F6 mAb pretreatment resulted in a significant reduction in mitochondrial ROS generation (Fig. 5F, J).

The mitochondrial respiratory chain complex, which is embedded in the inner mitochondrial membrane, organizes ATP production through an electron and proton transfers known as oxidative phosphorylation (OXPHOS). Although OXPHOS is effective in producing ATP, it also produces ROS, which is amplified in the presence of mitochondrial dysfunction [30]. To investigate the impairment of mitochondrial function, we assessed the activity of mitochondrial respiratory chain complexes. Upon 24 h of exposure to PAN, the activity of mitochondrial complex I, complex IV, and complex V in podocytes was significantly reduced, whereas pretreatment with the 5E5F6 mAb notably restored the activity of mitochondrial complex I, complex IV, and complex V (Fig. 5K).

**Fig. 6:**
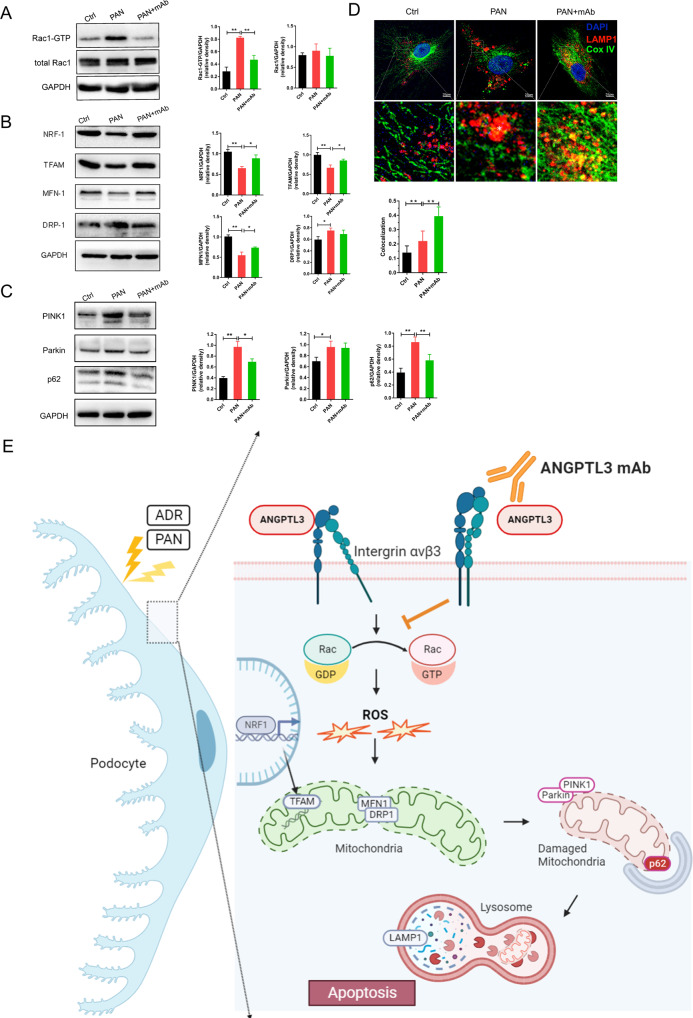
5E5F6 mAb treatment mitigates Rac1 activation and restores mitochondrial function. **A** Representative western blot gel documents of Rac1-GTP, Rac1 in podocytes with different treatments. Relative expression of Rac1-GTP, Rac1 in different groups. **B** Representative western blot gel documents of NRF1, TFAM, MFN-1and DRP1 in podocytes with different treatments. Relative expression of NRF1, TFAM, MFN-1and DRP1 in different groups. *p < 0.05, **p < 0.01 compared with the PAN group. **C** Representative western blot gel documents of PINK1, Parkin, p62 in podocytes with different treatments. *p < 0.05, **p < 0.01 compared with the PAN group. **D** Immunofluorescence analysis of podocytes in different treatment groups. Cox IV: mitochondrial marker, LAMP1: lysosome marker. Quantification of the colocalization area of Cox IV and LAMP1. *p < 0.05, **p < 0.01 compared with the PAN group. Asterisk: lysosome accumulation. **E** Illustration of treatment with 5E5F6 mAb improves mitochondrial dysfunction in podocyte damage. In response to ADR or PAN exposure, ANGPTL3 is highly expressed in podocytes, with the activation of integrin αvβ3 and Rac1 GTPase, and subsequent generation of ROS. ROS-induced mitochondrial impairment leads to apoptosis in podocytes. By blocking integrin αvβ3, Rac1 activation, and reducing ROS production, pretreatment with 5E5F6 mAb can reduce the effect of PAN on mitochondrial dysfunction and apoptosis in podocytes.

Rac1 activation increased the generation of ROS [31] which might be activated by integrin αvβ3 [26, 27]. The Rac1 signaling pathway is involved in the mitochondrial biogenesis [32, 33]. In comparison with the control group, Rac1-GTP (activated Rac1 form) levels were significantly elevated in PAN-treated podocytes, which were markedly reduced after pre-treatment with 5E5F6 mAb (Fig. 6A, Supplementary Fig. S8). In addition to ROS production and Rac1 activation, the expression of nuclear respiratory factor 1 (NRF-1) and mitochondrial transcription factor A (TFAM), the two key regulators of mitochondrial biogenesis, were down-regulated in the PAN-treated group, with the 5E5F6 mAb restoring the NRF1 and TFAM expression. Dynamin-related protein 1(Drp1) is the central regulatory protein for mitochondrial fission, while fusion proteins mitofusin1 (Mfn-1) and mitofusin 2 (Mfn-2) are required for the fusion reaction [12]. The elevated level of Drp1 and the declined level of Mfn-1 were observed by PAN treatment. Compared with the PAN-treated group, the 5E5F6 mAb had no effect on Drp1 expression, but significantly increased the expression level of Mfn1 (Fig. 6B, Supplementary Figs. S8, S9).

Damaged mitochondria need to be eliminated by a specific autophagy process known as mitophagy. We investigated the role of 5E5F6 mAb in podocytes with mitophagy induced by PAN. The levels of PTEN-induced putative kinase protein1 (PINK1), parkin (PARK2) and p62 were considerately higher in PAN-treated podocytes than those in the control group. The 5E5F6 mAb pretreatment could restore the elevated levels of PINK1 and p62, indicating a possible effect on mitophagy defect (Fig. 6C, Supplementary Fig. S9, S10). Additionally, the colocalization mitochondria with lysosomes was to assess the mitophagy-lysosome machinery. We found a lower proportion of mitochondria colocalizing with lysosomes in PAN-treated podocytes and more lysosome accumulation compared to those in the 5E5F6 mAb-treated cells (Fig. 6D). These data indicate that the defective mitophagy induced by PAN could be ameliorated by the 5E5F6 mAb.

## Discussion

Podocytes are differentiated epithelial cells of the glomerulus that play a critical role in the kidney filtration barrier. Podocyte damage inevitably leads to proteinuria and glomerular dysfunction [1]. Treatments for podocytes, on the other hand, are still quite restricted. In this study, we developed a monoclonal antibody against ANGPTL3-FLD, which showed efficacy in reducing proteinuria and restoring podocyte injury in vivo and in vitro partly through alleviating mitochondrial damage in podocytes.

ANGPTL3 belongs to a secretory protein family that has similarity with angiopoietins. It consists of two domains: the N-terminal coiled-coil domain (CCD) and the C-terminal fibrinogen-like domain (FLD). The N-terminal fragment CCD regulated the plasma levels of triglyceride by reversibly inhibiting the catalytic activity of LPL. The C-terminal fragment FLD binds to the integrin αvβ3 and participates in angiogenesis, which is comparable to angiogenin activity [34]. To investigate the possible effect of ANGPL3 target therapy, we first generated a mAb against human ANGPTL3-FLD sequence (5E5F6), which inhibited the binding of ANGPTL3-FLD and integrin β3. In ADR nephropathy mice, treatment with the 5E5F6 mAb reduced the proteinuria and led to a significant decline of podocyte apoptosis, ROS production and mitochondrial fragmentation. Overall, our findings suggested that the ANGPTL3-FLD mAb had a renal protective effect.

Integrins are essential for organ development, homeostasis, and the progression of kidney disease [35]. The activation of integrin αvβ3 on podocytes is documented in the pathogenesis of proteinuric kidney diseases such as FSGS and DN [36]. Blocking integrin αvβ3 activation by antagonist treatment can significantly reduce proteinuria, renal fibrosis, and subsequent disease progression in animal models [37]. So far, the only identified receptor for ANGPTL3 on the podocyte is integrin αvβ3. Our study demonstrated that the mAb against ANGPTL3-FLD (5E5F6) effectively blocked ANGPTL3-FLD binding to integrin β3 and reduced integrin αvβ3 activation in podocytes in vivo and in vitro. We also revealed that pretreatment with 5E5F6 mAb mimicked the protective effect of RGDyk, a selective Integrin αvβ3 inhibitor, by reducing the integrin αvβ3 activation and the ROS production. Integrin αvβ3 modulates ROS production via regulating small GTPases family molecules, and Rac1 is a key downstream component in the process [26, 27, 38]. Furthermore, we demonstrated that the pretreatment with the 5E5F6 mAb restored the Rac1-GTP activation in PAN-induced podocytes. Other inducible costimulatory ligands have recently been identified, however the RGD-binding integrin ligand that fine-tunes integrin function in podocytes has yet to be identified [39]. These data indicate that the activity of integrin and ANGPTL3 in the setting of podocytopathy needs be fine-tuned.

Mitochondrial dysfunction has been shown to be strongly linked to podocyte injury. Mitochondrial dysfunction occurs early in podocyte injury [28]. ROS is a major cause of mitochondrial dysfunction [28]. Moreover, Rac1 signaling affects the mitochondrial biogenesis-related proteins such as peroxisome proliferator-activated receptor-C coactivator-1a (PGC1a), NRF1 and TFAM [32]. Our findings demonstrated that the 5E5F6 mAb reduced mitochondrial damage in podocytes, as revealed by changes in mitochondrial morphology and function, in addition to reducing ROS generation and Rac1 activation. Mitochondrial homeostasis imbalance, mitochondrial biogenesis, morphology (fusion/fission) and degradation (mitophagy), all of which are closely related to mitochondrial dysfunction, have been implicated in the occurrence and progression of various glomerular diseases [12]. TFAM directly binds mtDNA, and NRF-1 is an important regulator of TFAM. The highly conserved dynamin-related GTPase dynamin-related protein 1 (Drp1), the mitofusins (Mfn1 and Mfn2) and optic atrophy 1 (OPA1) are the key mediators of mitochondrial dynamics [12]. PAN and ADR can induce mitochondrial fission in podocytes by inhibiting Mfn1 according to a previous study [40]. In the current study, the protein levels of NRF-1 and TFAM decreased, as well as the copy number of mtDNA declined following PAN treatment. NRF-1, TFAM and mtDNA copy number were all restored after pretreatment with the 5E5F6 mAb. The 5E5F6 mAb dramatically reduced the mitochondrial morphological changes by regulating MFN1 expression. When damaged mitochondrial cannot be restored by fission and fusion, they are generally eliminated through mitophagy, a critical component of mitochondrial quality control that identifies and tags severely damaged mitochondrial for clearance. It is mediated by PINK1 and Parkin system when the MMP is lost [12]. Dysregulated mitophagy has been previously shown to aggravate podocyte damage in proteinuric murine models. In the current study, we found an activation of the initial steps of mitophagy, as revealed by the elevated levels of PINK1 and Parkin in PAN-treated podocytes. However, the mitophagy process was disrupted, as indicated by the lack of p62 degradation, as well as the loss of co-localization of mitochondria and lysosome. The 5E5F6 mAb could improve the mitophagy degradation process by alleviating the impaired mitophagosome-lysosome fusion. These findings provide novel insights into the mechanisms by which ANGPTL3-FLD mAb mediates cytoprotective signaling.

Our study should identify additional questions. ANGPTL3 and integrin are not only expressed in podocytes; whether ANGPTL3 and integrin-β3 binding modulates the function of cells other than podocytes in the kidney remains to be determined. Our preliminary work in mice with ADR-induced nephropathy observed that whole body knock out of Angptl3 or podocyte-specific Angptl3 knock out had comparable benefits in reducing proteinuria and podocyte damage in (unpublished data), corroborating the findings of this study. Additionally, the monoclonal antibody used in this study requires further optimization. It is not a very high-affinity antibody. In the future, we plan to perform structural analysis to clarify the key binding locations of ANGPTL3-FLD and integrin, as well as to screen high-affinity antibodies to improve therapeutic efficacy.

In conclusion, we illustrated the therapeutic potential and mechanisms of anti-ANGPTL3 mAb in podocytopathy (Fig. 6E). By blocking the activation of integrin αvβ3 and Rac1 in PAN-induced podocyte damage, the anti-ANGPTL3-FLD mAb improved the fragmentation and dysfunction of mitochondria. Treatment of anti-ANGPTL3-FLD mAb restored podocyte injury, kidney lesions, albuminuria and metabolic abnormalities of ADR nephropathy mice. Thus, our study sheds insights into the role of anti-ANGPTL3-FLD mAb as a new prospective agent against NS.

## Supplementary information

Reproducibility Checklist

Supplementary files

## Data Availability

The datasets used and/or analyzed during the current study are available from the corresponding author on reasonable request.
