# Supplementary material for: Anti-ANGPTL3-FLD monoclonal antibody treatment ameliorates podocyte lesions through attenuating mitochondrial damage
Source: Cell Death Dis. 2022 Oct 13;13(10):867. doi: 10.1038/s41419-022-05313-7 (PMC9562403; doi:10.1038/s41419-022-05313-7)
Supplement: Supplementary file 2 — Supplementary files [file 41419_2022_5313_MOESM2_ESM.pdf]

Supplementary File

Figure S1: The thermostability of the anti-ANGPTL3-FLD monoclonal antibody. Tm of 5E5F6 is 64.65 °C.

Figure S2: The binding activity of ANGPTL3-FLD to Integrin αvβ3 was detected by ELISA assay. The minimum binding saturation concentration of ANGPTL3-FLD-biotin to Integrin αvβ3 is 0.16ug/ml.

Figure S3: 5E5F6 mAb protected against proteinuria in adriamycin nephropathy mice model. (a) BALB/c mice were injected with anti-ANGPTL3-FLD monoclonal antibody every four days in 4 weeks with different doses (10mg/kg, 20mg/kg, 40mg/kg). Proteinuria at 0 to 4 weeks. (b) Evaluation of albumin levels in the blood at the fourth week. (c) Evaluation of serum total cholesterol (T-CHO) levels at the fourth week. (d) Evaluation of serum creatinine at the fourth week. (e) Evaluation of albumin levels in the blood at the eighth week. (f) Evaluation of serum total cholesterol (T-CHO) levels at the eighth week. (g) Evaluation of serum creatinine at the eighth week. (h) Weight change of the mice from different groups was measured for 4 weeks. (i) Weight change of the mice from different groups was measured for 8 weeks.

Figure S4: Effect of 5E5F6 mAb treatment on renal lesions in ADR nephropathy mice model. Representative images of light microscopy of kidney sections of control mice, ADR nephropathy mice and anti-ANGPTL3-FLD monoclonal antibody-treated ADR nephropathy mice (ADR+mAb), stained with periodic acid–Schiff (PAS) in 4w. Original magnification: ×200 and ×400. Arrow head

indicates podocyte vacuole degeneration

Figure S5: Histology analysis of the major organs (brain, heart, liver, spleen, lung and kidney) stained with H&E. H&E stained images of brain, heart, liver, spleen, lung and kidney after treatment with 5E5F6 for 8weeks. Original magnification: ×200 and ×400.

Figure S6 The protein ladders of western blot for ANGPTL3, BAX, Bcl2, tubulin and GAPDH in different groups (n= 3).

Figure S7 The protein ladders of western blot for caspase 8, cleaved caspase 9 and cleaved caspase3 and GAPDH in different groups (n= 3).

Figure S8 The protein ladders of western blot for Rac1-GTP, total Rac1, NRF-1, TFAM and GAPDH in different groups (n= 3). .

Figure S9 The protein ladders of western blot for MFN-1, DRP-1, PINK1, Parkin and GAPDH in different groups (n= 3).

Figure S10 The protein ladders of western blot for p62 and GAPDH in different groups (n= 3).

Table S1: Sequence of primers for quantification of mitochondrial DNA content

gene	forward	reverse
Homo Cytb	TCACCAGACGCCTCAACCGC	GCCTCGCCCGATGTGTAGGA
Homo COⅡ	GGCACATGCAGCGCAAGTAGG	GGCGGGCAGGATAGTTCAGACG
18S rRNA	TTCGGAACTGAGGCCATGATT	TTTCGCTCTGGTCCGTCTTG

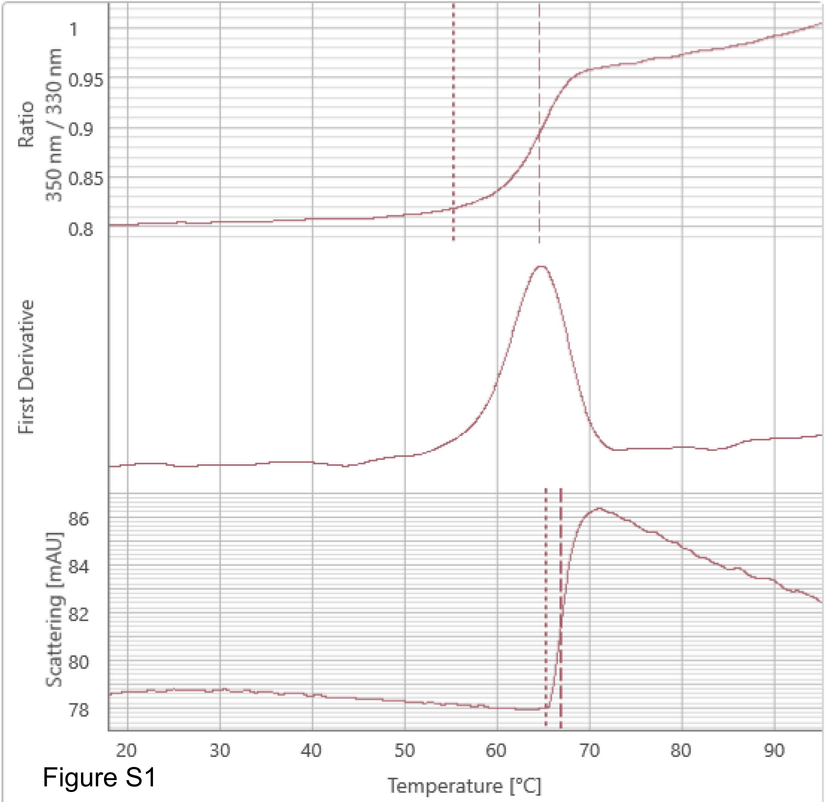

Ratio
350 nm / 330 nm
1
0.95
0.9
0.85
0.8
First Derivative
Scattering [mAU]
86
84
82
80
78
20
30
40
50
60
70
80
90
Temperature [°C]

Figure S1

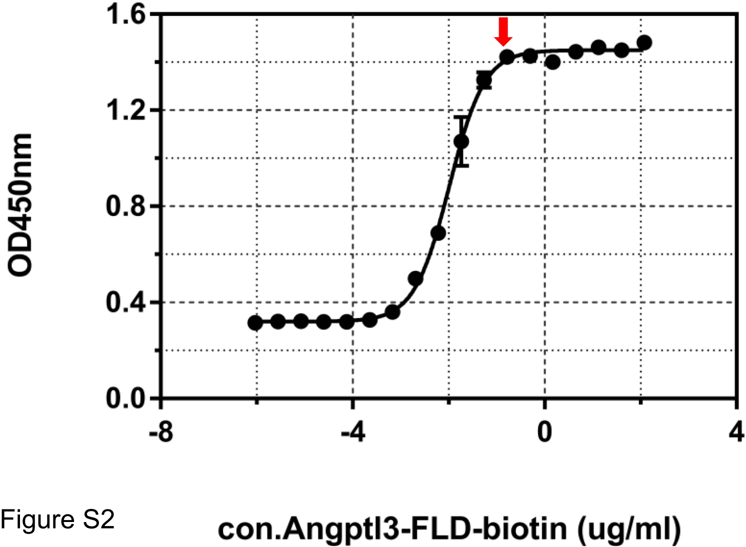

Figure S2

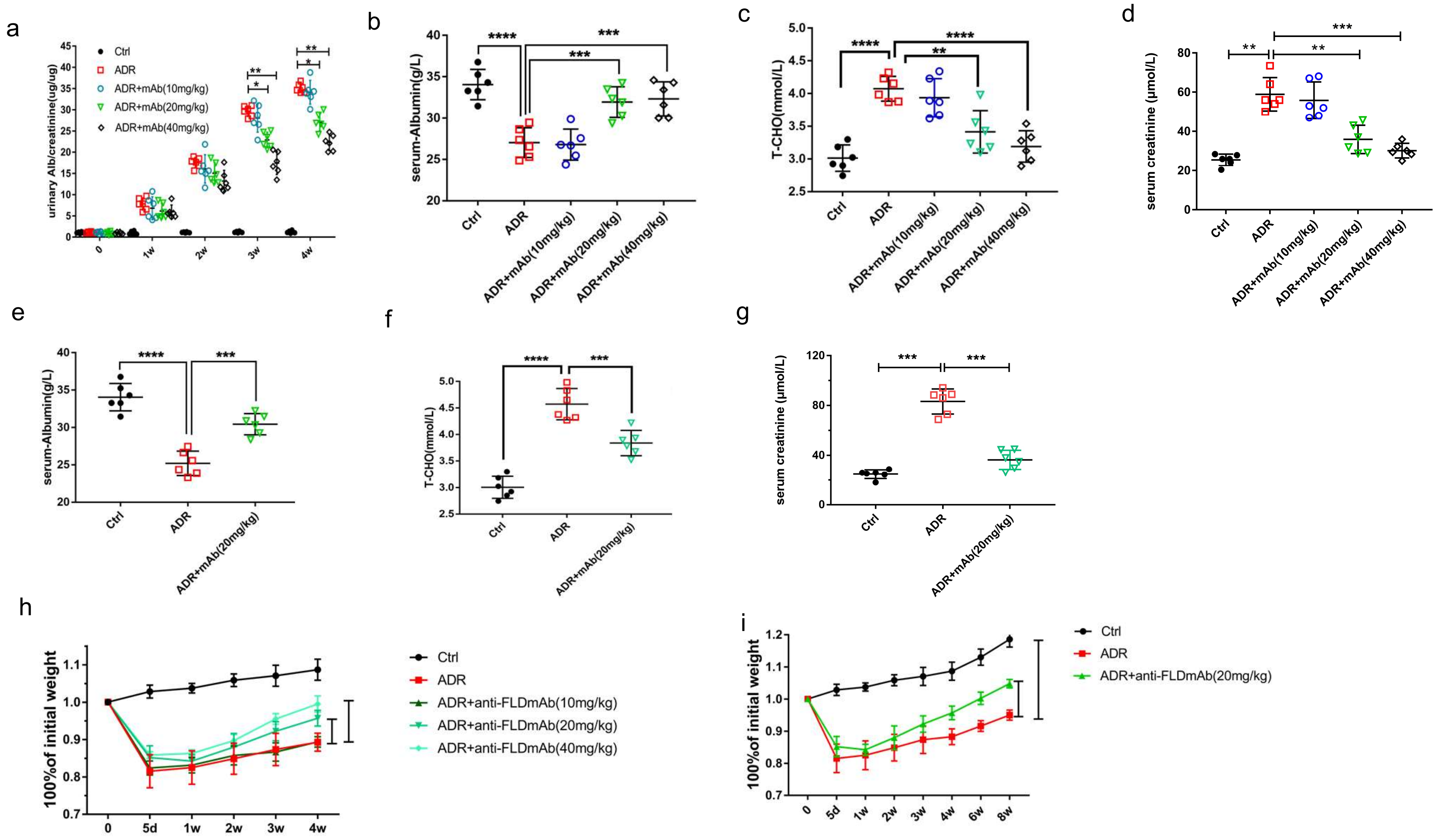

a
urinary Alb/creatinine(ug/ug)
Ctrl
ADR
ADR+mAb(10mg/kg)
ADR+mAb(20mg/kg)
ADR+mAb(40mg/kg)
b
serum-Albumin(g/L)
c
T-CHO(mmol/L)
d
serum creatinine (μmol/L)
e
serum-Albumin(g/L)
f
T-CHO(mmol/L)
g
serum creatinine (μmol/L)
h
100%of initial weight
ADR+anti-FLDmAb(10mg/kg)
ADR+anti-FLDmAb(20mg/kg)
ADR+anti-FLDmAb(40mg/kg)
i
100%of initial weight
ADR+anti-FLDmAb(20mg/kg)

Figure S3

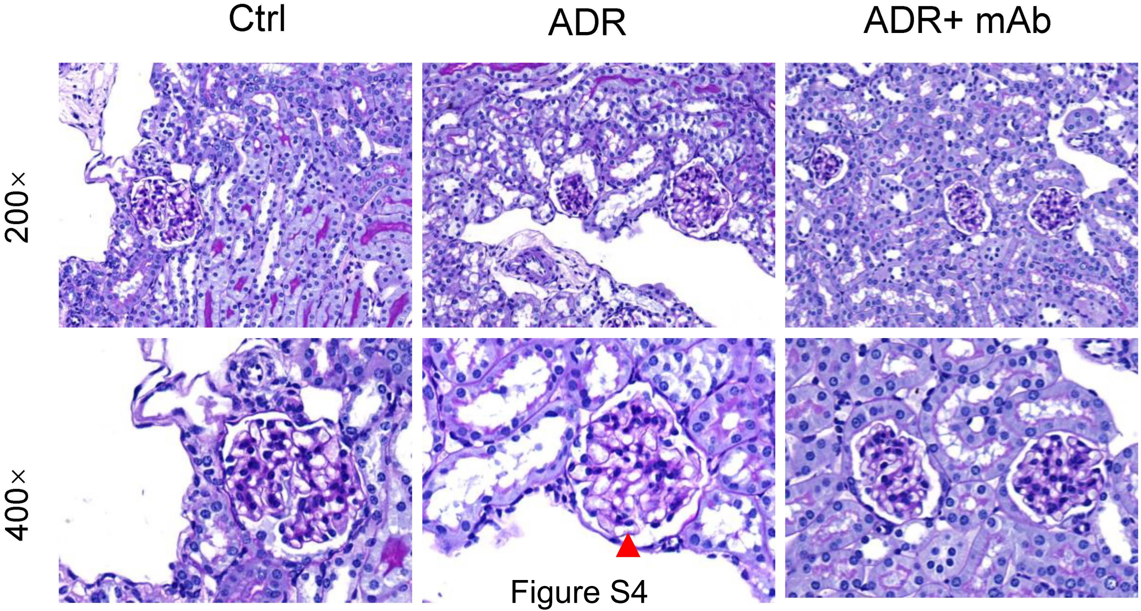

Figure S4

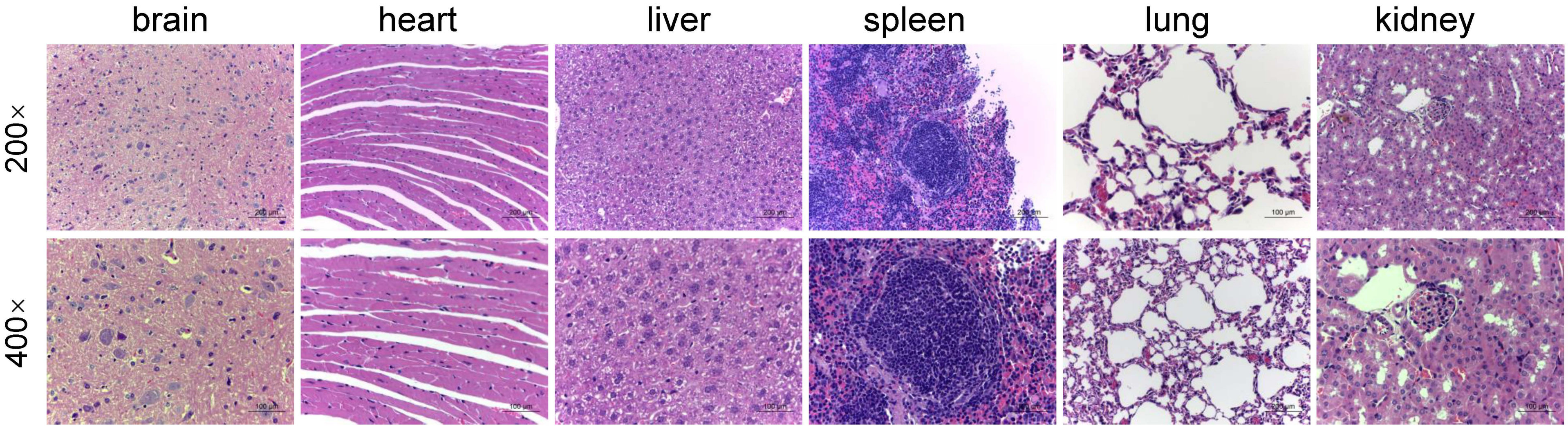

Figure S5

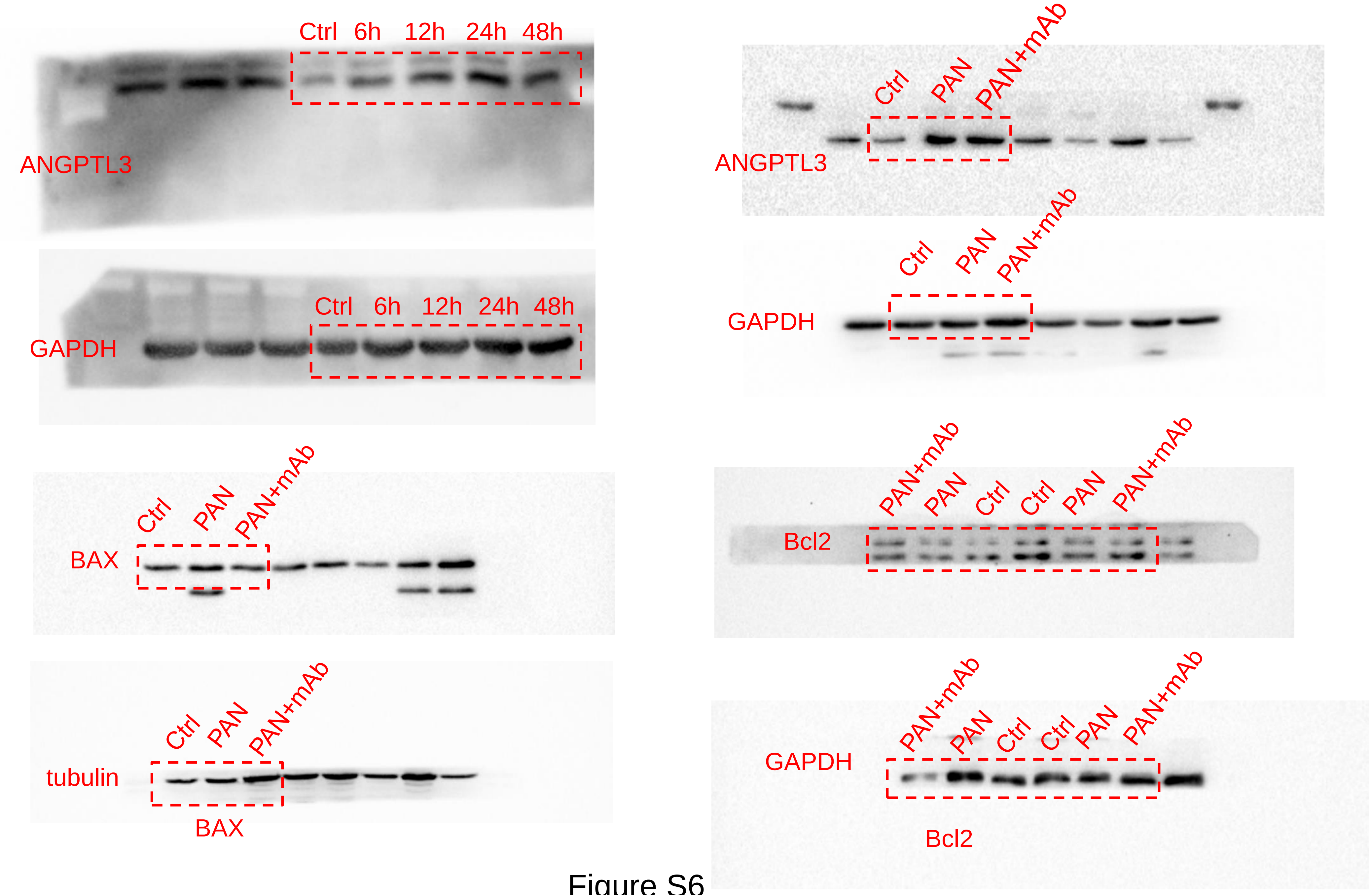
Ctrl 6h 12h 24h 48h
ANGPTL3
Ctrl 6h 12h 24h 48h
GAPDH
Ctrl
PAN
PAN+mAb
ANGPTL3
Ctrl
PAN
PAN+mAb
GAPDH
Ctrl
PAN
PAN+mAb
BAX
PAN+mAb
PAN
Ctrl
Ctrl
PAN
PAN+mAb
Bcl2
Ctrl
PAN
PAN+mAb
tubulin
BAX
PAN+mAb
PAN
Ctrl
Ctrl
PAN
PAN+mAb
GAPDH
Bcl2

Figure S6

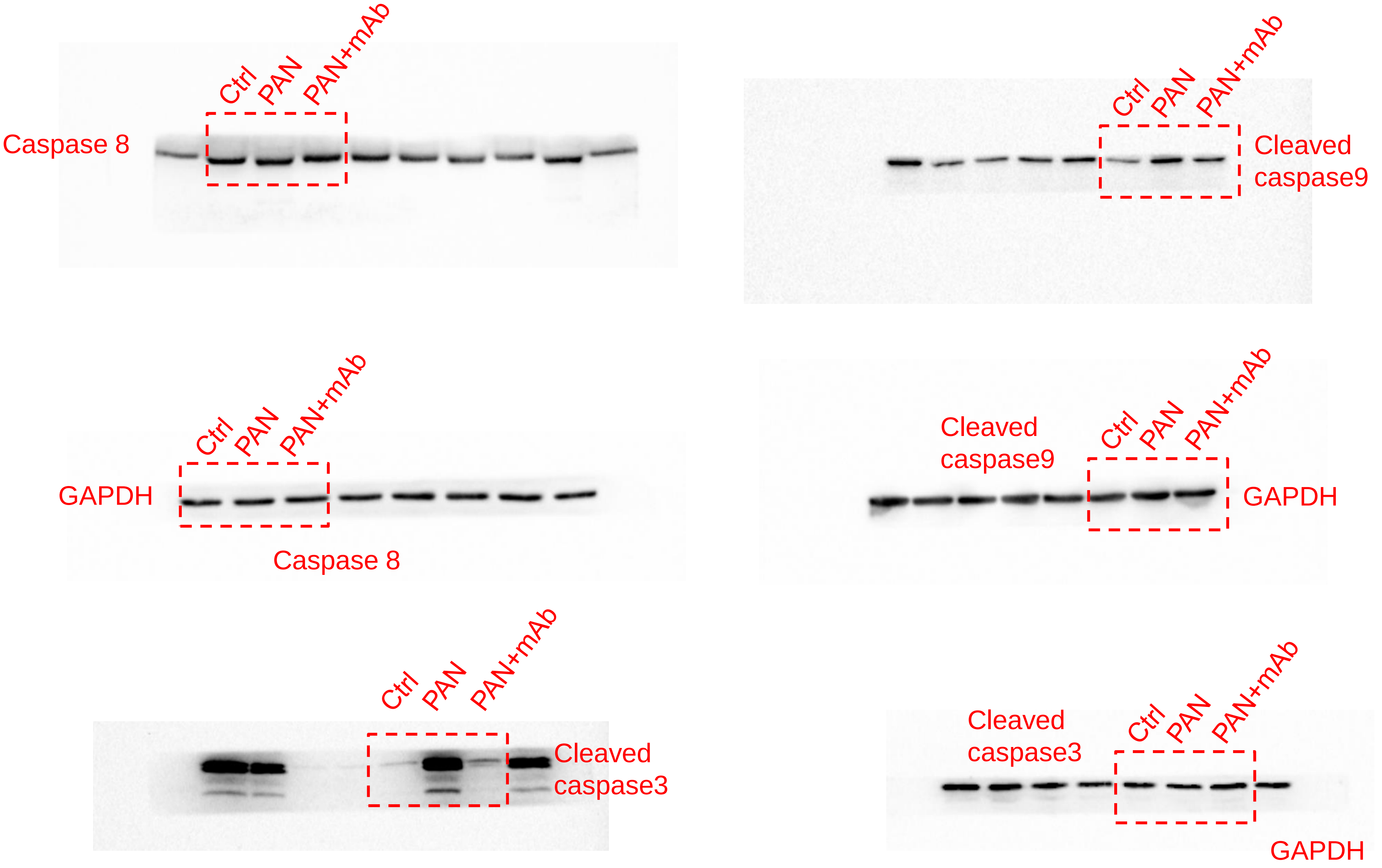
Ctrl
PAN
PAN+mAb
Caspase 8
Cleaved
caspase9
GAPDH
Caspase 8
Cleaved
caspase9
GAPDH
Cleaved
caspase3
Cleaved
caspase3
GAPDH

Figure S7

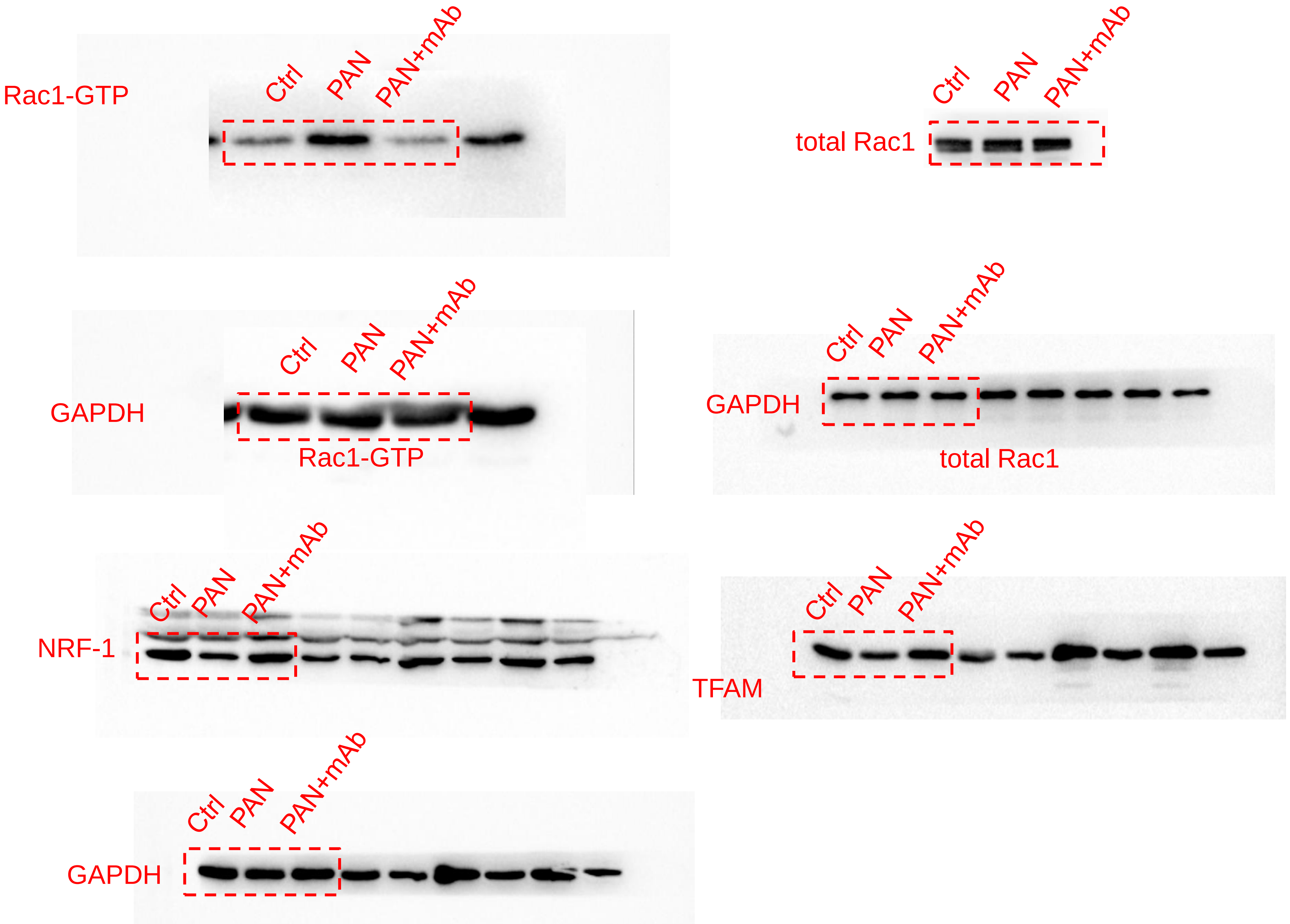
Rac1-GTP
Ctrl
PAN
PAN+mAb
total Rac1
Ctrl
PAN
PAN+mAb
GAPDH
Ctrl
PAN
PAN+mAb
Rac1-GTP
GAPDH
Ctrl
PAN
PAN+mAb
total Rac1
NRF-1
Ctrl
PAN
PAN+mAb
TFAM
Ctrl
PAN
PAN+mAb
GAPDH
Ctrl
PAN
PAN+mAb

Figure S8

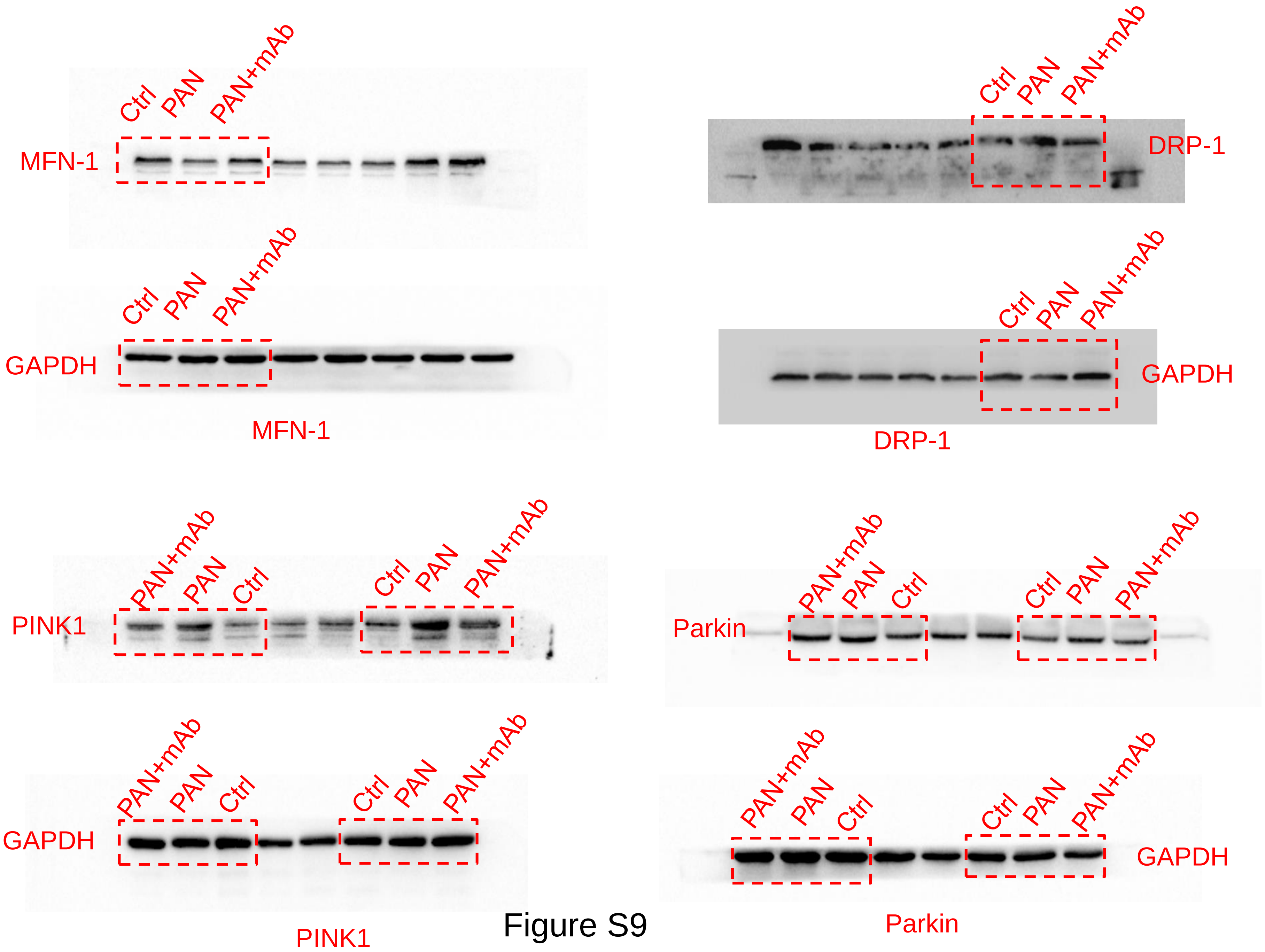
Ctrl
PAN
PAN+mAb
MFN-1
GAPDH
MFN-1
Ctrl
PAN
PAN+mAb
DRP-1
GAPDH
DRP-1
PAN+mAb
PAN
Ctrl
PINK1
GAPDH
PINK1
Parkin
GAPDH
Parkin

Figure S9

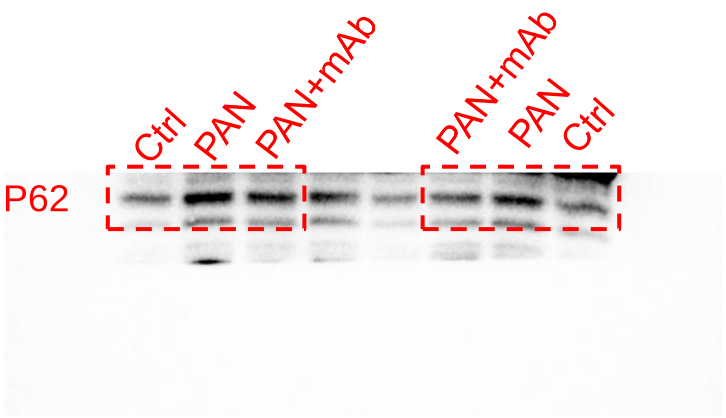
Ctrl
PAN
PAN+mAb
PAN+mAb
PAN
Ctrl
P62

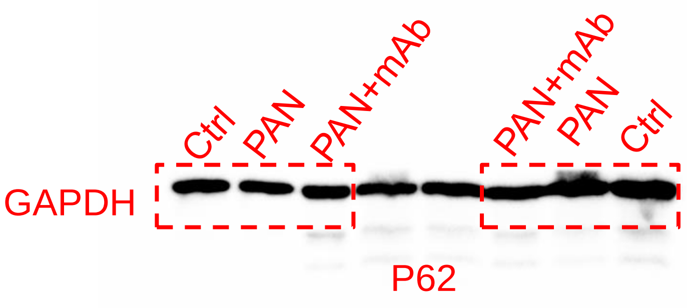
Ctrl
PAN
PAN+mAb
PAN+mAb
PAN
Ctrl
GAPDH
P62

Figure S10
